# Transmission potential of influenza A/H7N9, February to May 2013, China

**DOI:** 10.1186/1741-7015-11-214

**Published:** 2013-10-02

**Authors:** Gerardo Chowell, Lone Simonsen, Sherry Towers, Mark A Miller, Cécile Viboud

**Affiliations:** 1 Division of International Epidemiology and Population Studies, Fogarty International Center, National Institutes of Health, 31 Center Dr, MSC 2220, Bethesda 20892-2220, Maryland, USA; 2 Mathematical, Computational & Modeling Sciences Center, School of Human Evolution and Social Change, Arizona State University, 900 S. Cady Mall, Tempe 85287-2402, Arizona, USA; 3 Department of Global Health, School of Public Health and Health Services, George Washington University, 2175 K Street, Washington, DC 20037, USA

**Keywords:** Influenza A/H7N9, Transmissibility, Reproduction number, Spillover, Animal reservoir, Emerging infection, Influenza A/H5N1, Swine influenza, Transmission potential, China, Real-time estimation

## Abstract

**Background:**

On 31 March 2013, the first human infections with the novel influenza A/H7N9 virus were reported in Eastern China. The outbreak expanded rapidly in geographic scope and size, with a total of 132 laboratory-confirmed cases reported by 3 June 2013, in 10 Chinese provinces and Taiwan. The incidence of A/H7N9 cases has stalled in recent weeks, presumably as a consequence of live bird market closures in the most heavily affected areas. Here we compare the transmission potential of influenza A/H7N9 with that of other emerging pathogens and evaluate the impact of intervention measures in an effort to guide pandemic preparedness.

**Methods:**

We used a Bayesian approach combined with a SEIR (Susceptible-Exposed-Infectious-Removed) transmission model fitted to daily case data to assess the reproduction number (R) of A/H7N9 by province and to evaluate the impact of live bird market closures in April and May 2013. Simulation studies helped quantify the performance of our approach in the context of an emerging pathogen, where human-to-human transmission is limited and most cases arise from spillover events. We also used alternative approaches to estimate R based on individual-level information on prior exposure and compared the transmission potential of influenza A/H7N9 with that of other recent zoonoses.

**Results:**

Estimates of *R* for the A/H7N9 outbreak were below the epidemic threshold required for sustained human-to-human transmission and remained near 0.1 throughout the study period, with broad 95% credible intervals by the Bayesian method (0.01 to 0.49). The Bayesian estimation approach was dominated by the prior distribution, however, due to relatively little information contained in the case data. We observe a statistically significant deceleration in growth rate after 6 April 2013, which is consistent with a reduction in A/H7N9 transmission associated with the preemptive closure of live bird markets. Although confidence intervals are broad, the estimated transmission potential of A/H7N9 appears lower than that of recent zoonotic threats, including avian influenza A/H5N1, swine influenza H3N2sw and Nipah virus.

**Conclusion:**

Although uncertainty remains high in R estimates for H7N9 due to limited epidemiological information, all available evidence points to a low transmission potential. Continued monitoring of the transmission potential of A/H7N9 is critical in the coming months as intervention measures may be relaxed and seasonal factors could promote disease transmission in colder months.

## Background

An outbreak of novel A/H7N9 influenza virus infections rapidly unfolded in Eastern China, with the first laboratory-confirmed case identified in Shanghai on 31 March 2013 and a total of 132 laboratory-confirmed cases and 38 fatalities reported as of 3 June 2013 [1,2]. Although the number of new A/H7N9 cases has stalled since early May 2013, several features of this virus have heightened concerns for its pandemic potential and prompted an intense public health response from the Chinese authorities and international health organizations. Foremost, the rapid progression of new cases in urban centers in April 2013 and the severity of the disease have been worrisome. Although the exact route of transmission remains unclear, current evidence points to frequent spillovers from a yet-to-be-confirmed avian reservoir, suspected to involve poultry [3-6]. Although genetic analyses of the novel virus have revealed potential signs of adaptation to mammalian hosts [7], to date, sustained human-to-human transmission has not been established through contact tracing analysis [3,4] but cannot be ruled out. About 23% [4] of the A/H7N9 patients report having no prior exposure to live animals, underscoring the potential role of transmission by the environment, aerosols and undocumented contacts with infected individuals. Further, recent experimental studies indicate that the A/H7N9 virus is able to spread efficiently among ferrets via direct contact, although airborne transmission is less efficient [8].

A particular cause for concern is the fact that poultry infected with the A/H7N9 virus seem to exhibit relatively mild symptoms [9], which may extend the infectious period in this host. This is in stark contrast to highly pathogenic A/H5N1 influenza viruses, which typically kill poultry within a few days. Silent and undetected A/H7N9 infections in poultry increase the likelihood of zoonotic infections which, in turn, enhance the potential for acquisition of sustained human-to-human transmission properties.

Preliminary studies suggest a low incidence of A/H7N9 infection in chickens and pigeons in affected areas [1,5]. Nevertheless, live bird markets were preemptively closed and sick birds culled since 6 April 2013 in Shanghai and 16 April 2013 in Zhejiang, which may have slowed down the progression of the outbreak [10]. A quantification of the rate of viral transmission to humans and the effectiveness of intervention measures would be particularly useful to guide public health responses and provide a comprehensive risk assessment of the A/H7N9 threat.

The reproduction number, R, is a key epidemiological tool for assessing the transmission potential of an emerging infection and monitoring the likelihood of large-scale outbreaks. Estimates of R >1 signal the potential for an emerging pathogen to generate a major epidemic while R <1 indicates that transmission chains cannot be sustained in the population.

In the case of an emerging infection, obtaining near real time estimates of R is essential to guide intervention strategies. Bayesian estimation approaches [11-13] are naturally well-suited for situations where epidemiological data are gradually accumulating, due to their flexibility to incorporate prior information. In these approaches, prior information is sequentially updated as more complete outbreak data become available, providing posterior distributions of the epidemiological parameters of interest [11-13]. In contrast, more traditional 'epidemic curve fitting’ approaches have been typically used to provide retrospective estimates of *R* once the outbreak is over [14-19]. Alternative estimation approaches are based on detailed individual-level information on prior exposure to suspected animal reservoirs and/or contact with infected patients [20-22].

In this report, we estimate the transmission potential of the influenza A/H7N9 virus by relying on daily official notifications of laboratory-confirmed cases in mainland China. In particular, we focus on assessing whether the progression of the outbreak is consistent with unsustained human-to-human transmission dynamics in line with R <1 and whether intervention measures may have reduced transmission. Further, we compare R estimates for A/H7N9 with those for other zoonotic pathogens that have recently caused pandemic concern.

## Methods

### Data sources

**Figure 1:**
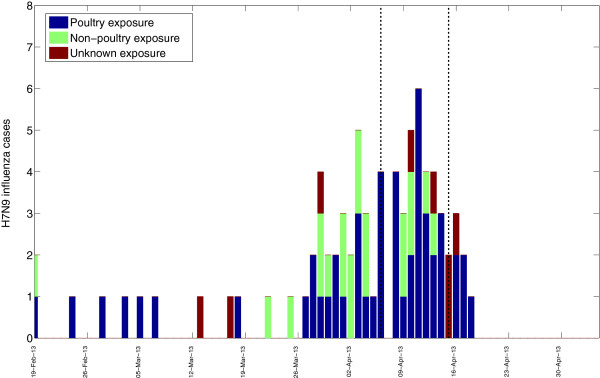
**Temporal incidence of laboratory-confirmed A/H7N9 influenza in the provinces of Shanghai and Zhejiang according to date of symptoms onset (n = 78).** Vertical dashed lines indicate the timing of the preemptive live bird market closure in Shanghai (6 April) and Zhejiang (15 April), respectively. Cases are color coded by exposure history.

We used official notifications of laboratory-confirmed A/H7N9 influenza cases reported in mainland China from 1 March to 20 May 2013 to the Chinese Center for Disease Control and Prevention (China CDC) through a national surveillance system. For each of the 130 cases, we obtained the exact date of symptoms onset, the province of residence and whether the patient had recent exposure to poultry or live bird markets. None of the records had missing information on residence location or onset date. We focused our analysis on Zhejiang and Shanghai provinces, where the majority (60%) of cases have been reported to date. A plot of the daily A/H7N9 epidemic curve is provided in Figure 1.

### Ethics

The dataset of laboratory-confirmed cases of avian influenza A H7N9 infection was part of a continuing public health investigation of an emerging outbreak and was, therefore, exempt from institutional review board assessment.

### Estimation of the reproduction number *R*

We adopted a sequential Bayesian framework combined with a susceptible-exposed-infectious-removed (SEIR) transmission model to estimate R for influenza A/H7N9 [11,12,23]. Here, the theoretical R value is a fixed (unknown) quantity, and R estimates are updated in a sequential Bayesian framework as data accumulate over time. This approach was previously applied to study the dynamics of the A/H5N1 influenza outbreaks in Asia [23], the 1918 influenza pandemic in San Francisco, USA [12], and the 2009 A/H1N1 influenza pandemic in China [24]. In this model, the population is assumed to be well-mixed. Susceptible individuals (S) come in contact with infectious individuals (I) and progress to the exposed stage (E) with an average latency period of k^-1^ days. Exposed individuals (E) then progress to the infectious stage (I), with an average infectious period of γ^-1^ days. Both the latent and infectious periods are assumed to be exponentially distributed.

This model assumes that all A/H7N9 cases originate from human-to-human transmission and, hence, provides an upper bound on the transmissibility of A/H7N9. We also conducted simulation studies to assess the performance of this approach in the situation of an emerging pathogen, where most human cases are due to spillover events originating from exposure to an animal reservoir or the environment, and human-to-human transmission is limited [See Additional file 1].

In the Bayesian SEIR approach, we use a relationship that is directly applicable to time series data as it expresses the expected number of new cases over the time period τ (for example, τ = 1 day) as a function of the number of cases in the previous time period, given with prior epidemiological information. The relation follows from a standard SEIR model [11,23]:

(1) $$E\left[C\left(t+\tau \right)\right]=b\left(R,\gamma ,\kappa \right)C\left(t\right),$$

where E[C(t + τ)] is the expected number of new cases at time t + τ, b(R, γ, κ) defines the progression of cases, γ^-1^ and κ^-1^ are the infectious and latent periods, respectively, and C(t) is the observed number of new cases at time t. The progression operator is given by:

(2) $$b\left(R,\gamma ,\kappa \right)=\text{exp}\left[\lambda_{+}\tau \right],$$

Where λ_+_ is the dominant eigenvalue derived from linearization of the SEIR model around disease-free equilibrium, following [15]

(3) $$\lambda_{+}=\frac{\left(\kappa +\gamma \right)}{2}\left[-1+\sqrt{1+\frac{\kappa \gamma }{{\left(\kappa +\gamma \right)}^{2}}\left(R-1\right)}\right].$$

In this approach, both the latent and infectious periods (1/γ, 1/κ) are fixed and, hence, the only parameter to be estimated is R. We made two different assumptions for the latent and infectious periods to illustrate a short infection process consistent with seasonal influenza [25] (k^-1^ = 1.5 days and γ-^1^ = 1.5 days, so that the generation interval is 3.0 days) and a longer infection process in line with descriptions of the prolonged course of A/H7N9 infections in humans (k^-1^ = 3 days, γ-^1^ = 3 days, so that the generation interval is 6.0 days) [4,5,26].

### Bayesian inference of the reproduction number *R*

We formulate the model in discrete time probabilistic form to account for the discrete nature of the influenza case data and estimate the distribution of R using Bayes’ theorem.

The distribution of new incident cases *C*(*t* + τ) follows:

(4) $$C\left(t+\tau \right)∼R\left[C\left(t+\tau \right)\leftarrow C\left(t\right)\left|R\right)\right]$$

which states that *C*(*t* + τ) only depends on the number of new cases at the previous time point *C*(*t*), given *R*. Using Bayes’ theorem, the updated posterior distribution of R at day t + τ follows:

(5) $$P\left[\left(R\right|C\left(t+\tau \right)\leftarrow C\left(t\right)\right]=\frac{P\left[C\left(t+\tau \right)\leftarrow C\left(t\right)\left|R\right)\right]P\left[R\right]}{P\left[C\left(t+\tau \right)\leftarrow C\left(t\right)\right]}$$

where the denominator is a normalization factor. Hence, Equation (5) defines the sequential Bayesian estimation scheme, where the posterior probability distribution of *R* can be used as a prior to generate a posterior distribution at the next time step.

We have to set an initial prior on R to initialize the sequential approach at *t* = 0, which can reflect any *a priori* knowledge of the disease. Based on preliminary R estimates derived from the exposure history of A/H7N9 patients (see below), we assumed normal distributions centered around 0.2 (SD = 0.2) and 0.5 (SD = 0.2) as initial priors for R; both distributions were left-truncated at 0. We also consider a more extreme prior center at R = 1 in Additional file 1.

To compute numerically the posterior of R at each daily iteration, we use Equation 5, relying on the posterior from the previous day as the new prior, following [11,12]. The posterior R distribution was evaluated using 1,000 discrete bins between 0 and 1.5.

#### Simulation studies

We carried out simulation studies to evaluate the performances of the Bayesian sequential estimation method in the context of an emerging pathogen. Specifically, we simulated A/H7N9 influenza outbreaks using a modified SEIR transmission process including different levels of human-to-human transmission (as measured by R) together with spillover events originating from a hypothetical reservoir. We varied the true R in the range 0.1 to 2.0 and modeled spillover events as a constant daily rate of new infections arising from exposure to the reservoir (α, in the range 1 to 10 infections per day). We used the model to simulate daily outbreak data, applied the Bayesian estimation method to these data, and confronted the estimated *R* with the true R [see Additional file 1].

These simulations were designed to gauge the level of error associated with neglecting transmission from environmental or animal sources in our main Bayesian estimation approach, and also to assess the sensitivity of R estimates to prior distribution assumptions, under different epidemiological scenarios.

#### Variance on case series of A/H7N9 influenza

The SEIR transmission model imposes a requirement on the mean of A/H7N9 cases, but variance can be modeled in a more flexible manner. Because we are dealing with disease count data, the most general choice is the Poisson distribution, where the mean equals the variance. As sensitivity analysis we considered a Negative Binomial distribution which allows for greater variance and better accounts for over-dispersed data, and assumed the variance to be twice the mean.

#### Estimating the impact of live bird market closures

To estimate the impact of live bird market closures in the most affected provinces of Shanghai and Zhejiang, we fit an exponential curve with intrinsic growth rate *r* to the daily case time series in the pre-intervention period (before 6 April). We used a Negative Binomial log likelihood fit to account for over-dispersion in case counts. The 95% confidence intervals on the growth rate were determined from the range of values of *r* that yield log L = log L_max - s^2/2 where s = 1.96, and L_max is the value of the likelihood at the best-fit value of *r*[27]. Using the exponential model fit up to 6 April, we forecasted the expected number of A/H7N9 cases in subsequent weeks. We confronted the progression of reported cases past 6 April against that predicted by the pre-intervention model as an indication of the effectiveness of control measures.

#### Reproduction number estimates based on individual-level exposure data

As a complementary method to estimate the R for influenza A/H7N9, we used an approach recently developed by Cauchemez *et al*. for zoonotic infections [20]. In this approach, R = 1-p, where p is the estimated proportion of infected patients arising from direct contact with the A/H7N9 reservoir (scenario 1 in [20]). This approach provides a conservative upper bound on R as it assumes that case detection probability is independent of cluster allocation (while in general, once an index case is identified, other infections in the family are more likely to be detected). This is a reasonable approach when human-to-human transmission is low [20].

An alternative approach to estimate R relies on the average size of chains of human-to-human transmission, as R can be estimated by dividing the number of secondary infections occurring within clusters by the number of primary cases with a direct link to the reservoir [21]. Although there is uncertainty in the exposure history of A/H7N9 patients, the nature of the reservoir of this virus, and cluster sizes and frequency, we can use R estimates based on exposure and contact information [22,23] to set the initial prior distributions for R in our Bayesian estimation scheme.

Finally, we provide a comparative review of the transmission potential of emerging zoonoses using both individual-level contact tracing and exposure data and transmission model fitting approaches, with a focus on avian influenza A/H5N1, swine influenza A/H3N2v, seasonal and pandemic influenza, Nipah virus and severe acute respiratory syndrome (SARS).

## Results

### Influenza A/H7N9 epidemic curves

**Figure 2:**
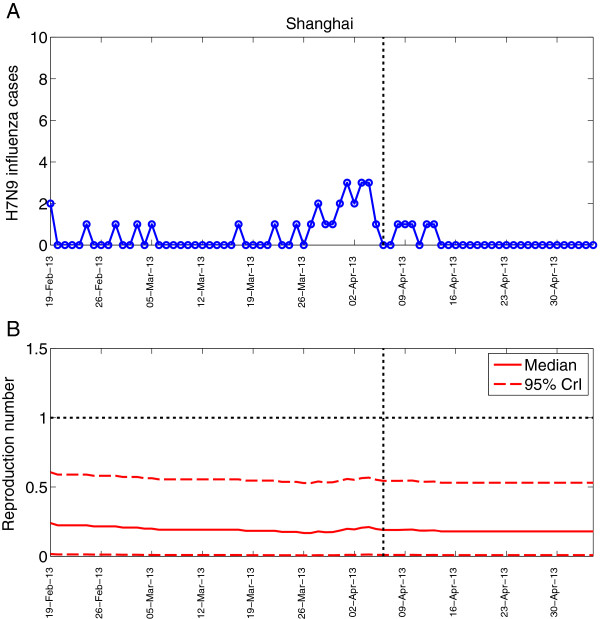
**Epidemic curve and sequential Bayesian estimation of the distribution of *R *for the A/H7N9 influenza outbreak in Shanghai, China. A)** Daily number of laboratory-confirmed A/H7N9 influenza cases by date of symptoms onset. Vertical dashed lines indicate the timing of the preemptive live bird market closures in Shanghai (6 April). **B)** Evolution of R estimates as data accumulate over time, assuming a prolonged serial interval of six days (latent period, k^-1^ = 3 days and infectious period, γ-^1^ = 3 days). Median *R* (solid red line) and 95% credible intervals (dashed red lines) are shown. The horizontal dotted line indicates the threshold at R = 1, above which large epidemics are expected to occur. R, reproduction number.

**Figure 3:**
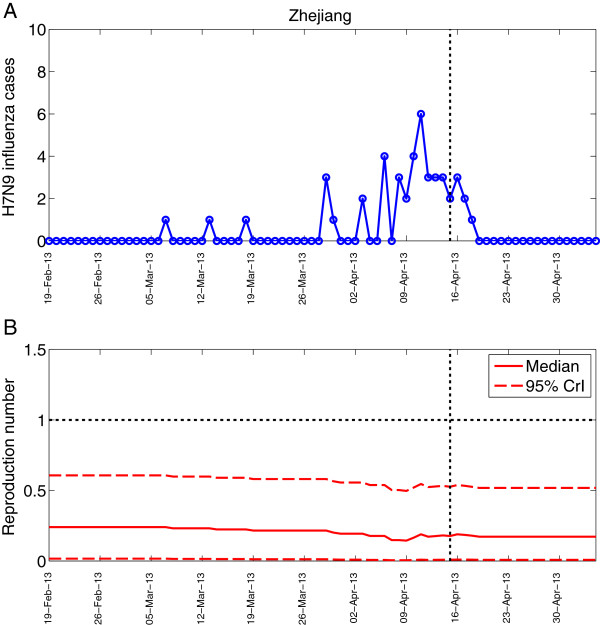
**Epidemic curve and sequential Bayesian estimation of the distribution of *R *for the A/H7N9 influenza outbreak in Zhejiang province, China. A)** Daily number of laboratory-confirmed A/H7N9 influenza cases by date of symptoms onset. Vertical dashed lines indicate the timing of the preemptive live bird market closures in Zhejiang (15 April). **B)** Evolution of R estimates as data accumulate over time, assuming a prolonged serial interval of six days (latent period, k^-1^ = 3 days and infectious period, γ-^1^ = 3 days). Median R (solid red line) and 95% credible intervals (dashed red lines) are shown. Horizontal dotted line indicates the threshold at R = 1, above which large epidemics are expected to occur. R, reproduction number.

Figure 1 illustrates the course of the A/H7N9 epidemic by date of symptom onset in Zhejiang and Shanghai provinces from 19 February to 26 May 2013. Overall, 71.4% (50/70) of the influenza A/H7N9 cases reported in these provinces were associated with exposure to poultry and/or live bird markets (Figure 1). Figures 2 and 3 present the progression of the outbreak separately in Shanghai (n = 33) and Zhejiang (n = 45). The incidence accelerated around 27 March 2013 in Shanghai (the first of three consecutive days with non-zero cases), and approximately two weeks later in Zhejiang on 8 April 2013.

### Reproduction number estimates based on the Bayesian sequential approach

**Figure 4:**
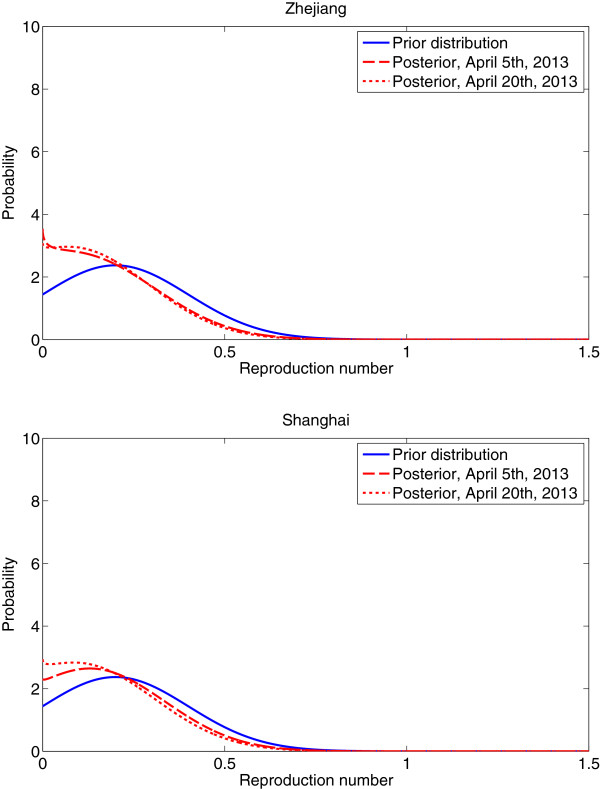
**Comparison of prior and posterior distributions for the reproduction number, R, associated with the A/H7N9 outbreak in Zhejiang (top) and Shanghai (bottom), using the sequential Bayesian SEIR estimation method.** Sequentially obtained posterior distributions are based on data up to 15 April, immediately prior to the first closure of live bird markets, and up to 20 April, two weeks into the intervention period. We assume a serial interval of six days (latent period k^-1^ = 3 days and infectious period γ-^1^ = 3 days). The initial prior for R is a normal distribution left-truncated at 0 and centered at 0.2 (SD = 0.2). SEIR, susceptible-exposed-infectious-removed.

The Bayesian sequential estimation approach revealed that the Shanghai and Zhejiang A/H7N9 data were most consistent with a R around 0.1, with broad 95% credible intervals (0.01 to 0.49) excluding 1 (Table 1). The progression of updated R estimates as data accumulate over time is shown in Figures 2 and 3 for each province; there was no significant change in estimated R as the outbreak progressed from February to May 2013 in either location. The prior and posterior distributions for R are compared in Figure 4 and reveal a moderate change as the outbreak progresses, suggesting that there is relatively limited information in the A/H7N9 case data.

**Table 1:** Estimates and 95% credible intervals of the reproduction number, R, for the A/H7N9 influenza outbreak in China

**Parameters**	**R estimate (95% CI)**	
	**Zhejiang**	**Shanghai**
(k^-1^ = 3 days and γ-^1^ = 3 days)	0.13 (0.01 to 0.46)	0.15 (0.01 to 0.47)
(k^-1^ = 1.5 days and γ-^1^ = 1.5 days)	0.11 (0.003 to 0.42)	0.17 (0.01 to 0.49)

R estimates based on the sequential Bayesian estimation SEIR method, prior to the start of control interventions on 6 April 2013.

### Sensitivity analyses and simulation studies

A sensitivity analysis on the prior distribution for *R* confirmed that there was high uncertainty in the posterior estimates of *R* [see Additional file 1: Figure S1]. However, the posterior mean of R and upper 95% credible interval remained below the epidemic threshold (R = 1) as epidemiological data accumulated, no matter the prior. Further, estimates were robust to assumptions regarding variance in case count data [see Additional file 1: Figure S2].

Next, we simulated outbreak data illustrating the spread of an emerging infection, where human cases originate from both human-to-human transmission and direct contact with a hypothetical reservoir. Simulations indicate that the Bayesian estimation approach tends to overestimate R, especially when the true R is low and spillover events are frequent [see Additional file 1: Figure S3]. However, the upper bound of the credible interval of the Bayesian approach was trustworthy, as it remained below 1.0 whenever the true R <0.6. Further, case data from Shanghai and Zhejiang suggest that the reported rate of spillover transmission from the reservoir was in the order of approximately one daily infection in the pre-intervention period, which is in the lower (and more favorable) range of our simulations.

Importantly, simulations show a substantial change between prior R distribution (centered at 0.2, as in our main analysis) and posterior R distributions, when the true R is above 0.6 [see Additional file 1: Figure S3]. This suggests that if the true R was above 0.6 for A/H7N9, we would have detected a greater change in posterior distribution than we did in the observed outbreak data. Finally, our simulation studies indicate that the proportion of A/H7N9 patients arising from human-to-human transmission is approximately equal to R, when 0.1 ≤ R ≤0.9 [see Additional file 1: Figure S4].

Additional sensitivity analyses considering longer latent and infectious periods did significantly change *R* estimates (Table 1). Similarly, assuming a Negative Binomial to model over-dispersion in A/H7N9 case data did not significantly affect our estimates [see Additional file 1: Table S1, Figure S2].

### Impact of intervention measures

**Figure 5:**
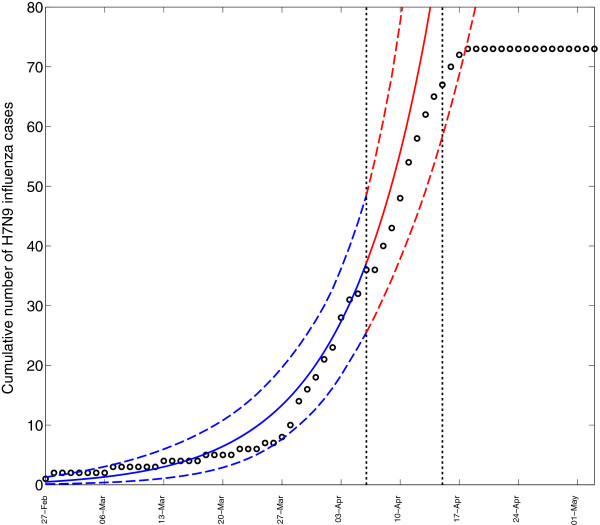
**Predicted progression of cumulative laboratory-confirmed A/H7N9 cases in the provinces of Shanghai and Zhejiang (n = 73 cases) according to dates of symptoms onset, in the absence of interventions (solid blue line).** Dashed blue lines represent 95% confidence intervals. Predictions are based on an exponential model fit to the progression of reported cases from the end of February to 6 April, prior to live bird market closures, and using a negative binomial distribution to account for over-dispersion in case counts. Shown in red is the prediction of the model fit past 6 April. Black dots indicate the progression of reported A/H7N9 cases. Vertical dashed lines indicate the timing of the preemptive live bird market closures in Shanghai (6 April) and Zhejiang (15 April), respectively.

To gauge the impact of preemptive bird market closures, we analyzed temporal trends in cumulative daily A/H7N9 incidence by fitting an exponential curve to data for the combined provinces of Shanghai and Zhejiang, in the pre-intervention period 1 March to 6 April (Figure 5). Our results indicate a statistically significant (non-zero) intrinsic growth rate at 0.101 case/day (95% CI: 0.070 to 0.143). The model can be used to predict disease incidence past 6 April had there been no intervention. We note a deceleration in growth rate of observed cases past 6 April, outside of confidence bounds predicted by the pre-intervention model (Figure 5). In particular, the model identifies a statistically significant departure from predicted incidence by 18 April and throughout the end of the study period. A similar pattern was obtained by using nationally aggregated incidence data instead of province-level data [see Additional file 1: Figure S5].

### Estimates of the reproduction number for A/H7N9 using alternative approaches

As a complementary analysis, we present R estimates for A/H7N9 based on alternative approaches relying on individual-level information on prior exposure and contacts with infected patients [20,21].

Among the 130 A/H7N9 patients reported by 26 May 2013, in mainland China, three family clusters ranging in size from two to three were identified, with onset dates between 11 February and 21 March [see Additional file 1, Table 2; see also [4,28]]. Of the 130 cases, 67% reported a history of poultry exposure (88/122; eight have unknown exposure information), including 47% of patients who visited live bird markets (37/79, 51 unknown). Based on the proportion of new infections presumed to arise directly from the poultry reservoir [20], we can estimate R is approximately 1–0.67 = 0.23 (Table 2). An upper bound for R is provided by assuming a stricter definition of exposure solely based on exposure to live bird markets (the hypothetical reservoir), which yields an upper R estimate of 1–0.47 = 0.53.

**Table 2:** Comparison of reproduction number estimates for the A/H7N9 influenza viruses, other emerging zoonoses with pandemic potential, and human influenza viruses

**Outbreak**	**R estimate**	**Source and method**
A/H7N9 outbreak		
Avian influenza A/H7N9- 2013, China	0.1 (95% CrI: 0.01 to 0.49)	This study; Bayesian approach from [11]
Avian influenza A/H7N9- 2013, China	0.03 to 0.05	This study; exposure-based approach from [20]
Avian influenza A/H7N9- 2013, China	0.28 (95% CI: 0.11 to 0.45)	Analysis of cluster size distribution from [22]
Other zoonotic influenza viruses		
Avian influenza H5N1 -2003 to 2006, SE Asia and Egypt/Turkey	0.29	Cluster size distribution approach [21]; data from [29]
Avian influenza H5N1 – 2004 to 2006; SE Asia and Egypt/Turkey	0.52 to 0.54	[11] Bayesian approach
Swine influenza H3N2v - 2011, USA	0.5 to 0.74	Exposure-based approach [20]; data from [30]
Human influenza viruses		
1918 A/H1N1 influenza pandemic	1.8 to 5.4	[16,18,31,32] Various approaches
1957 A/H2N2 influenza pandemic	1.5	[33] growth rate
1968 A/H3N2 influenza pandemic	1.5	[33] growth rate
2009 A/H1N1 influenza pandemic	1.2 to 3.1	[17,34-40] Various approaches
Seasonal influenza	1.3	[41,42] growth rate
Other zoonotic viruses		
Nipah virus, Malaysia, 1990s	0.05 to 0.08	Exposure-based approach [20]; data from [43]
Nipah virus, Bangladesh, 2000s	0.48 to 0.51	Exposure-based and cluster size distribution approaches [20]; to data from [21]
SARS virus, Singapore, Hong Kong, 2003	2.2 to 3.6	[15,44] Epidemic model fitted to case series during the pre-intervention period

SARS, severe acute respiratory syndrome.

An alternative R estimate is provided by the average distribution of secondary chains of transmission. If we assume that all three A/H7N9 clusters represents one spillover event (primary case) followed by one to two serial transmission events, we obtain R = 4/126 = 0.03. Inclusion of one additional suspected cluster of size two identified by contact tracing [see Additional file 1] results in a slightly higher estimate of R = 5/126 = 0.04. Hence, information on individual-level exposure and cluster size distribution indicates that R is approximately 0.03 to 0.53, consistent with the broad range of uncertainty obtained in the Bayesian approach.

### Comparison of transmissibility estimates between influenza A/H7N9 and other zoonotic viruses

Table 2 presents a comparison of R for the A/H7N9 influenza virus, zoonotic influenza viruses, seasonal and pandemic influenza viruses and other viruses of pandemic concern. Estimates are based on a variety of approaches, including transmission model fitting methods and individual-level exposure history approaches (See Additional file 1 for details).

We compiled R estimates for zoonotic influenza viruses that episodically cause human infections, in particular for avian-origin A/H5N1 and swine-origin A/H3N2v. Estimates in the range 0.52 to 0.54 have been proposed for A/H5N1 in Thailand and Indonesia, based on a Bayesian approach similar to that used here [11]. Using the ratio of secondary infections to primary cases [29], we obtain R approximately 0.29 in this period of relatively intense H5N1 activity.

The H3N2v swine-origin influenza virus has recently become a cause of concern in the US, especially in the context of agricultural fairs in 2011 and 2012. Information on the proportion of patients with direct exposure to swine [30] suggests that R is approximately 0.67. Other approaches making more complex assumptions about surveillance intensity and over-dispersion in the distribution of secondary cases indicate that R is approximately 0.5 to 0.74 [20].

In the case of seasonal and pandemic influenza outbreaks, model-fitting approaches reveal that R is 1.3 on average for seasonal outbreaks [41,42] and 1.2 to 5.4 for pandemic viruses, with the highest estimates associated with the lethal 1918 pandemic [16-18,31-40] (Table 2).

Nipah virus is another emerging viral zoonosis worth comparing to influenza A/H7N9 (Table 2). Early outbreaks in Malaysia in the late 1990s were associated with low transmission potential, as most cases had direct exposure to swine, with *R* = 0.05 to 0.08 [43]. In contrast, more recent outbreaks in Bangladesh in 2001 to 2007 were characterized by a higher frequency of human-to-human transmission, with R approximately 0.51 [20,21]. A similar estimate was obtained by analyzing the cluster size distribution [21].

Table 2 also provides data for the SARS outbreak in 2003, with an estimated R in the range 2.2 to 3.7 based on fitting transmission models to the progression of weekly cases before intervention took place [15,44]. Hence, taken together, the influenza A/H7N9 virus currently has relatively low estimated transmission potential relative to other zoonotic viruses, although confidence intervals are broad.

## Discussion

We have provided near real-time estimates of the transmission potential of the emerging A/H7N9 influenza outbreak in China by applying different methodological approaches to official notifications of laboratory-confirmed cases. Although there is relatively limited information in the A/H7N9 case data at this point, all available evidence points to R estimates well below 1.0 in Shanghai and Zhejiang provinces, where the majority of cases have been reported. Instead, a deceleration in growth rate in mid April is consistent with the effectiveness of preemptive live bird market closures initiated in early April. Comparison between A/H7N9 and other zoonotic threats suggests a relatively low transmission potential relative to that of other avian or swine influenza viruses and recent Nipah viruses, although further data are necessary to confirm this result.

Our Bayesian SEIR estimation approach assumes that all infections originate from human-to-human transmission and, hence, yields 'worst-case scenario’ R estimates. Our estimation framework was robust to assumptions about the duration of the infectious and latent periods, whether we considered a short serial interval characteristic of seasonal influenza [25] or a prolonged disease course more consistent with early case descriptions [4,26]. In contrast, the Bayesian approach was very sensitive to assumptions regarding the prior distribution of R, which dominated the inference process. Using assumptions reasonably guided by information on prior patient exposure and the frequency of family clusters, this approach indicates a R well below the epidemic threshold (R = 1.0) in Eastern China. Further, simulation studies suggest that if the true R was above 0.6, we would see a greater shift from prior to posterior distributions than seen in the A/H7N9 data, confirming the low transmission potential of this virus.

Alternative estimation approaches based on individual level contact tracing and prior exposure suggest a range of R of 0.03 to 0.53, in line with a recent modeling study analyzing the cluster size distribution of A/H7N9 cases [22]. These low R estimates are consistent with the results of intense efforts by the Chinese health authorities to monitor contacts of infected cases, which have so far revealed only limited instances of secondary transmission [4]. While the occurrence of three (perhaps four) family clusters of A/H7N9 cases is consistent with short chains of human to human transmission, these clusters do not rule out exposure to common environmental or animal sources. Taken together, information from contact surveys [4] and available R estimates are consistent with a predominance of spillover events from a hypothetical reservoir.

We observed a reduction in the growth rate of H7N9 cases in mid to late April, coinciding with the closure of live bird markets in Shanghai, Zhejiang and large Chinese cities in response to the evolving outbreak. The deceleration in the growth rate was significant in our data as early as 18 April, a period when the effectiveness of these measures was still being debated [45]. Our model is ill-equipped, however, to predict the progression of the outbreak in the coming weeks if intervention measures are relaxed [46], as information is lacking on the residual prevalence of A/H7N9 in poultry populations in China. Further, we cannot rule out a subsequent rise in A/H7N9 transmission potential in the coming months, as seasonal factors could affect virus prevalence in the (presumed) avian reservoir and promote avian-to-human and possibly human-to-human transmission [47,48].

We have provided transmissibility estimates for influenza A/H7N9 and other zoonoses using several approaches, which rely on very different assumptions. The Bayesian SEIR model-fitting approach is based on the progression of case incidence; our analyses suggest that currently available A/H7N9 data provide relatively limited information, so that the inference process is heavily dependent on the prior (see also more extreme priors in Additional file 1: Figure S6). This likely stems from the small number of A/H7N9 cases available for study (n = 70 in the two main provinces), in part resulting from the low transmission potential of A/H7N9. Simulations were particularly helpful in showing that if the true R was above 0.6, then we would have most likely identified a shift in the posterior distribution. The lack of observed shift is further evidence that R is low and most likely below 0.6.

In the context of subcritical outbreaks (R <1), alternative methods based on contact tracing and exposure information are attractive, although they depend heavily on prior knowledge of the ecology of the disease. These methods rely on estimates of the proportions of cases arising from human-to-human transmission versus direct exposure to the reservoir [20,21] and, hence, assume that the reservoir is well known and that onset dates and serial intervals can be accurately determined. Further, methods relying on cluster size distribution are more sensitive to reporting schemes than growth rate methods (for example, if clusters are more likely to be reported once a family member is infected) [22].

Information regarding the reservoir of A/H7N9 and the natural history of this disease is still limited, as would be the case for any emerging zoonosis with limited prior experience. It is intriguing that 23% of A/H7N9 cases do not report any prior contact with poultry (suggesting R is approximately 0.23), and yet clusters are extremely infrequent (suggesting R closer to 0). These conflicting findings could be reconciled with additional information on the prevalence of asymptomatic infections; unfortunately, recent serological information is currently lacking. Overall, all R estimation methods tend to produce high uncertain ranges for A/H7N9. In a similar context, early estimates of the transmissibility of the MERS-CoV virus using a related approach were relatively broad, with confidence intervals ranging between 0.5 and 1.1 [49]. A quantitative comparison of the performances of these approaches would be useful in the future as these methods are increasingly applied to characterize the pandemic potential of emerging pathogens (see also [22]).

This study is subject to limitations. First, A/H7N9 incidence could be underreported. However, serological surveys conducted at the end of 2012 in China and Vietnam revealed low levels of prior infections [50,51]. Moreover, influenza-like-illness surveillance suggests that A/H7N9 infection was an uncommon cause of illness in any age group during March and April 2013 in the most affected areas of China [52]. Our estimates are resilient to underreporting issues as long as the observed case series closely tracks the true course of the outbreak. If case detection had improved over time with increased detection capabilities, this would have artificially quickened the progression of reported cases and, in turn, spuriously overestimated the epidemic growth rate and R. Hence, because of likely increased sampling intensity as the outbreak progressed, we can view our R estimates as upper bounds of the true value.

Second, we have used a simple model to estimate R, relying on a SEIR transmission model typically used for human diseases, while in fact there is likely very little transmission between humans. Our simulations suggest that in the context of frequent spillover events arising from a reservoir, our estimates of R are inflated (consistent with providing worst-case scenarios of the true human-to-human transmission potential of A/H7N9). However, our approach accurately predicts whether an emerging pathogen remains below the critical epidemic threshold (R <1). A more refined approach could integrate more information regarding the hypothetical reservoir and the probability of contacts with humans, and could estimate the relative contribution of each component to overall disease transmission. The yet unresolved nature of the reservoir of A/H7N9 and its ecology hampers the calibration of such models.

Third, our model assumes homogeneous mixing, which may not be valid. We have focused on province-specific data, which provides a better approximation of well-mixed populations than nationally-aggregated data, especially as most cases arose from large cities (especially Shanghai). Still, there could be residual spatial heterogeneity, which may artificially decrease the estimated R. Overall, our very generic model only requires information on the date of symptoms onset and could be applicable to a variety of emerging infections that include spillovers from a putative reservoir and human-to-human transmission.

## Conclusion

In conclusion, we have shown that the available epidemiological data on influenza A/H7N9 are consistent with subcritical transmission potential below R = 0.6 in the first three months of virus circulation in Shanghai and Zhejiang provinces, suggesting infrequent human-to-human transmission events. A decline in the growth rate of influenza A/H7N9 cases in April 2013 highlights the beneficial impact of live bird market closures. The estimated transmission potential of A/H7N9 appears lower than that of other zoonotic threats, although uncertainty remains important due to limited statistical information in the available data. Our proposed approach could be useful to quantify the progression of the outbreak and the impact of control measures in the coming months and help monitor the pandemic potential of this emerging pathogen in near real-time.

## Abbreviations

R: Reproduction number; SARS: Severe acute respiratory syndrome; SEIR: Susceptible-exposed-infectious-removed.

## Competing interests

The authors declare they have no competing interests.

## Authors’ contributions

GC and CV designed the experiments/the study. GC, LS, ST, MM and CV analyzed the data. GC and CV wrote the first draft of the paper. GC, LS, ST, MM and CV contributed to the writing of the paper. All authors read and approved the final manuscript.

## Pre-publication history

The pre-publication history for this paper can be accessed here:

http://www.biomedcentral.com/1741-7015/11/214/prepub

## Supplementary Material

Additional file 1 Supplementary information. Click here for file
