# Supplementary material for: Transmission potential of influenza A/H7N9, February to May 2013, China
Source: BMC Med. 2013 Oct 2;11:214. doi: 10.1186/1741-7015-11-214 (PMC3851127; doi:10.1186/1741-7015-11-214)
Supplement: Additional file 1 — Supplementary information. [file 1741-7015-11-214-S1.doc]

**Transmission potential of influenza A/H7N9, February-May 2013, China: Supplementary information**

Gerardo Chowell^1,2^, Lone Simonsen^1,3^, Sherry Towers^2^, Mark A. Miller^1^, Cécile Viboud^1^

^1^ Division of International Epidemiology and Population Studies, Fogarty International Center, National Institutes of Health, Bethesda, MD, USA

^2^ Mathematical, Computational & Modeling Sciences Center, School of Human Evolution and Social Change, Arizona State University, Tempe, AZ, USA

^3^ Department of Global Health, School of Public Health and Health Services,

George Washington University, Washington DC, USA

1. **Simulation studies to evaluate the performances of the Bayesian** *R* **estimation method in the context of an emerging pathogen**

We carried out simulations to evaluate the performances of the Bayesian sequential SEIR estimation method to evaluate *R* in the context of an emerging pathogen, particularly for A/H7N9 influenza. Specifically, we simulated A/H7N9 influenza outbreaks using a modified SEIR transmission process with varying levels of human-to-human transmission intensity (as measured by the reproduction number R) combined with spillover events from a hypothetical reservoir. We varied the true reproduction number R between 0.1 and 2.0 and modeled spillover events as a constant daily rate of introduction of new infections arising from exposure to the reservoir, α, where α varied from 1-10 cases per day (in keeping with the observed progression of the outbreak in the pre-intervention period in China).

For this purpose, we used a modified version of the simple stochastic SEIR type (susceptible-exposed-infectious-recovered) transmission model . In this model, the population is divided in 5 categories: Susceptible (S), Exposed (E), Symptomatic and Infectious (I) and recovered (R) individuals. The total population size (N=1 Million) is assumed constant and initially completely susceptible to emerging viral infection. We also assume that the population is well-mixed. That is, each individual has the same probability of having contact with any other individual in the population.

Susceptible individuals infected with the virus enter the latent period (category E) at the rate (β I(t)/N + α) where β is the mean transmission rate per day and α is the daily rate of new infections arising from exposure to the reservoir. Latent individuals progress to the infectious class at the rate $\kappa$ (1/$\kappa$ is the mean latent period). Infectious individuals recover at the rate γ, where the mean infectious period is given by 1/γ. Recovered individuals are assumed to be fully protected for the duration of the outbreak. The deterministic equations of the transmission process described above are given by:

dS/dt = – βS I/N - α

dE/dt = β S I/N + α – $\kappa$ E

dI/dt = $\kappa$ E - γ I

dR/dt = γ I

**2 Stochastic outbreak realizations**It is particularly important to consider stochastic rather than deterministic simulations due to higher stochasticity of outbreak progression at low values of transmission potential R. For this purpose, we generated 50 stochastic epidemic realizations of the model described above through a Poisson simulation approach , for each pair of values of true R and α Poisson Simulation is used to generate stochastic simulations from systems where the events happen randomly and independently. That is, the number of events during a time interval dt becomes Poisson-distributed. Hence, flows into and out of compartments (state variables) during dt can be generated as integer-valued and Poisson-distributed random process .

Each simulated outbreak realization was initialized by a number of spillover events defined by parameter α.

**3 Epidemiological parameters**

For a given value of the true reproduction number (R) in the range 0.1-2.0, the mean transmission rate β was set according to the relationship β = R γ . We assumed a mean generation interval of 6 days (k^-1^ = 3 days and $\gamma$^-1^ = 3 days) in line with descriptions of the prolonged course of A/H7N9 infections in humans . However, our simulation results varied little when assuming a short infection process consistent with seasonal influenza (k^-1^ = 1.5 days and $\gamma$^-1^ = 1.5 days) .

**4 R estimation using the sequential Bayesian approach**

For each of the 50 outbreaks simulated under each epidemiological scenario (characterized by a combination of true R and α) , we applied our SEIR Bayesian approach detailed in the main text to estimate R (ie, we use an SEIR model that neglects transmission from the reservoir). We compared the estimated posterior distribution of R with the true R value. We used a normal distribution left-truncated at 0 and centered at 0.2 (SD=0.2) as the initial prior for the reproduction number, no matter the value of true R used in simulations.

**5 Results of simulation studies**

The performance of the estimation method as a function of α and the true reproduction number R in the range 0.1-0.9 is shown in Figure S3. Simulation results indicate that our Bayesian R estimation approach tends to overestimate the reproduction number when the contribution of human-to-human transmission is low (ie, when the true R<0.6), but that the approach provides an appropriate upper bound for R that is below 1.0 in this situation. The Bayesian sequential method also tends to derive more accurate estimates of the reproduction number when transmission from the reservoir is infrequent, ie at low values of α.  Perhaps not surprisingly, the Bayesian approach provided accurate estimates of R whenever true R>1, ie most cases originate from human-to-human transmission. This has also been demonstrated in earlier studies .

Our simulation studies also indicate that the proportion of human-to-human transmission events among the total number of A/H7N9 cases is given approximately by R, whenever R<1. That is, the proportion of human-to-human transmission events was only weakly dependent on α, for the range of considered α (Figure S4).

A similar property can be used to estimate R from contact tracing and exposure information data, following R=1-p, where p is the proportion of cases with direct link to the reservoir .

**6. Additional information on A/H7N9 clusters.**

Of the 130 cases of A/H7N9 infections reported until May 26, 2013 by China CDC, three family clusters ranging in size from 2-3 have been identified (Table 2). The first cluster involves the initial laboratory-confirmed case reported in Shanghai, his father and brother (onset dates Feb 11 and Feb 19); the second cluster involves a middle-aged couple from Shanghai (onset dates Mar 27 and Apr 1), and the third a father and daughter from Jiangsu province (onset dates Mar 8 and Mar 21) . A fourth potential cluster involving two boys who were neighbors was also reported in Beijing (onset date Apr-11), although one boy was asymptomatic and only identified through intense contact tracing . The asymptomatic boy is not included in our official dataset. We excluded the 4^th^ cluster (and asymptomatic boy) from our main analysis, following recommendations in . Inclusion of this 4^th^ cluster would not change R estimates obtained by any of the methods considered in this study.

**7. Additional information on R estimates for other emerging pathogens.**

We compiled R estimates for zoonotic influenza viruses that episodically cause human infections, in particular for avian-origin A/H5N1 and swine-origin A/H3N2v. Estimates in the range 0.52-0.54 have been proposed for A/H5N1 in Thailand and Indonesia, based on a Bayesian approach similar to that used here . Family aggregation of A/H5N1 infections is not uncommon, as 36 clusters of size 2-8 have been identified among 261 cases reported during 2003-2006 in South East Asia, Egypt and Turkey . Individual-level exposure information on these 261 patients is lacking, but if we rely on the ratio of secondary infections to primary cases , we obtain R ~0.29 (58/203) in this period of relatively intense H5N1 activity.

The H3N2v swine-origin influenza virus has recently become a cause of concern in the US, especially in the context of agricultural fairs in 2011-2012. Of the 12 cases of the more transmissible H3N2v virus variant carrying the 2009 pandemic A/H1N1 matrix and reported in 2011, 4 have had direct contact with swine (33%, ), indicating that R~0.67. A related approach making more complex assumptions about surveillance intensity and overdispersion in the distribution of secondary cases suggests that R~0.5-0.74 .

In the case of human pathogens such as seasonal and pandemic influenza viruses, methods based on individual-level data break down, and R is best estimated by fitting transmission models to population-level epidemic curves. These approaches indicate that R is 1.3 on average for seasonal outbreaks and 1.2-5.4 for pandemic viruses, with highest estimates associated with the lethal 1918 pandemic (Table 2).

Nipah virus is another emerging viral zoonoses worth comparing to influenza A/H7N9 due to its pandemic potential (Table 2). Early outbreaks in Malaysia in the late 1990s were associated with low transmission potential, as 92-98% of cases were associated with direct exposure to swine, resulting in *R* = 0.05–0.08 . In contrast, more recent outbreaks in Bangladesh in 2001-2007 are characterized by a higher frequency of human-to-human transmission, with 49% of the 122 cases reporting direct exposure to swine, resulting in R~0.51 . A similar estimate is obtained by analyzing the cluster size distribution, with 29 secondary infections originating from 60 primary cases, indicating that R~0.48 .

Table 2 also provides data for the SARS outbreak in 2003, with estimated R in the range 2.2-3.7 based on fitting epidemic models to the growth rate in weekly cases before intervention took place in Asia and elsewhere .

**Supplementary Figures and Tables**

**Figure S1**. Sensitivity of R estimates to assumptions regarding prior distributions, for the H7N9 outbreak in Shanghai and Zheijiang provinces. Shown are posterior distributions for R (red curves), estimated using the sequential Bayesian estimation method prior to the closure of live bird markets on April 6^th^ assuming a serial interval of 6 days (k^-1^ = 3 days and $\gamma$^-1^ = 3 days). Sequentially obtained posterior distributions are based on data up to April 5^th^, immediately prior to the first closure of live bird markets, and up to April 20^th^, 2 weeks into the intervention period. The initial prior on R is a normal distribution left-truncated at 0 and centered at 0.5 (SD=0.2; blue curve). Compare to Figure 4, based on a prior centered at 0.2.

**
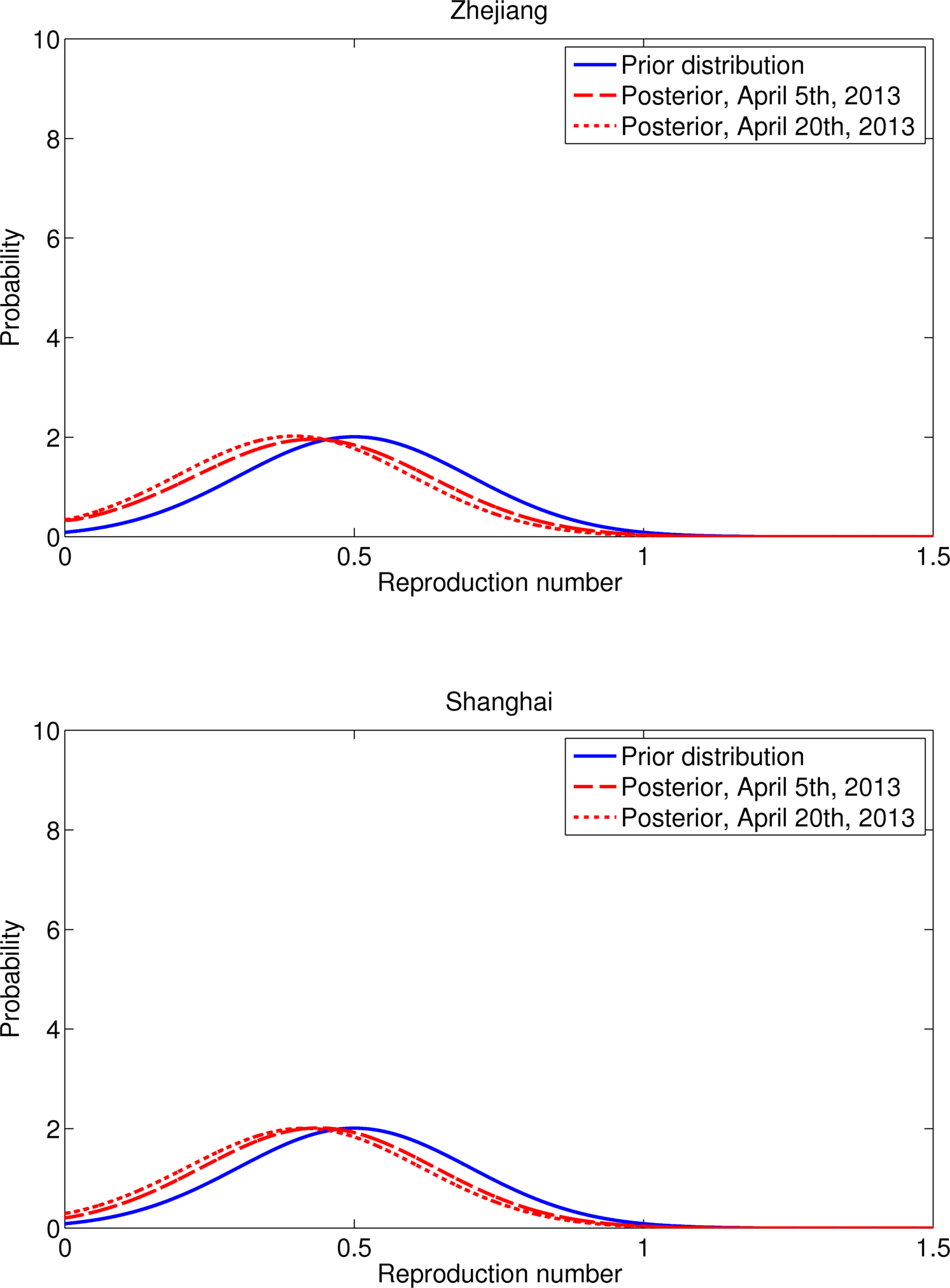
**

**Figure S2**. Sequential Bayesian estimation of *R* for the H7N9 influenza outbreak in Shanghai, China, assuming a Negative Binomial distribution to model variability in case counts. A) Daily number of laboratory-confirmed H7N9 influenza cases by date of symptoms onset in Shanghai, China. The vertical dashed lines indicate the timing of the preemptive live bird market closures in Shanghai (April 6). B) Evolution of R estimates as data accumulate over time assuming a prolonged serial interval of 6 days (k^-1^ = 3 days and ^-1^ = 3 days); median *R* (solid red line) and 95% CrIs (dashed red lines) are shown. Horizontal dotted line indicates the epidemic threshold value at R=1 above which large epidemics are expected to occur.

**
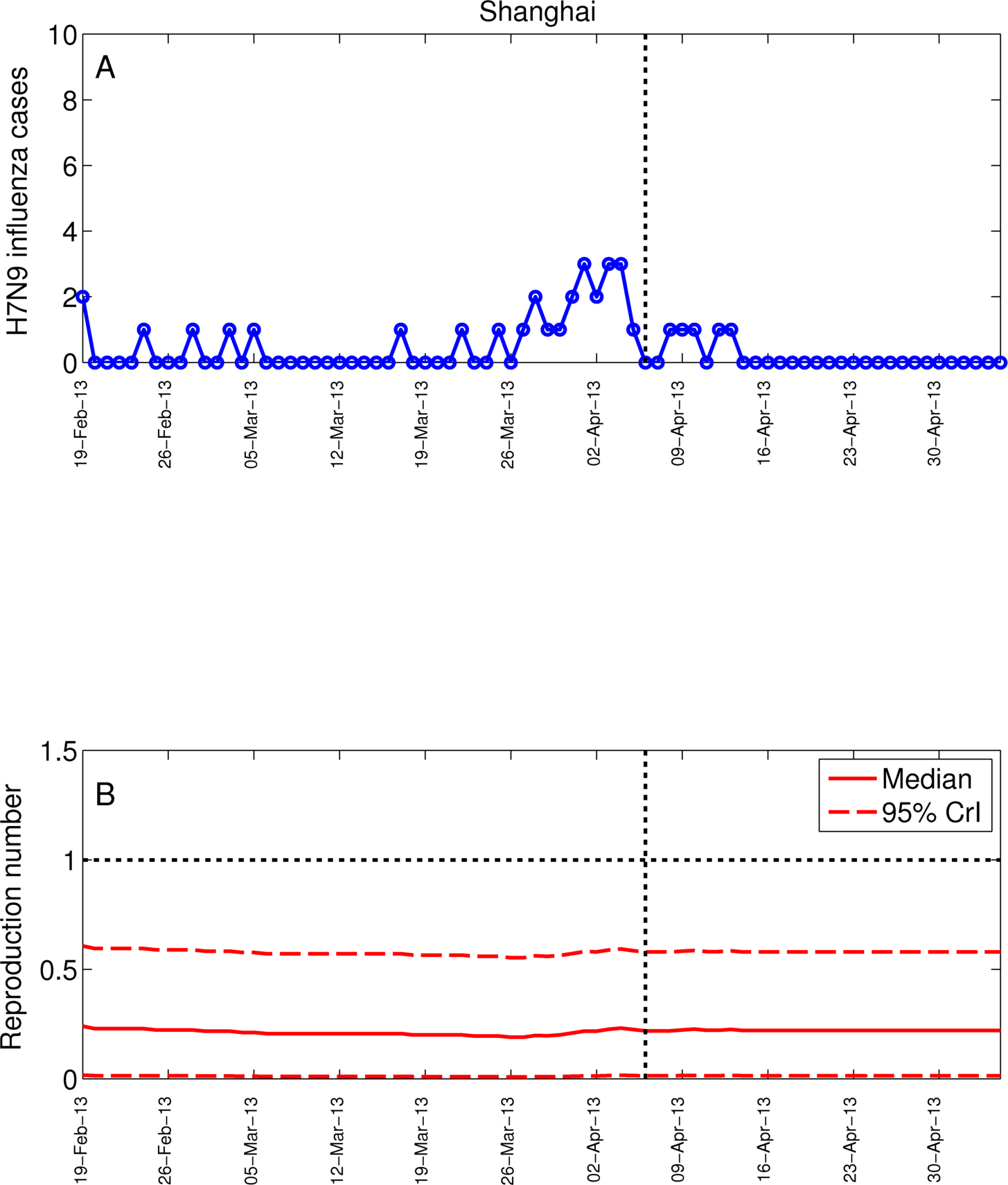
**

**Figure S3: Discrepancy in R estimates derived from the Bayesian SEIR estimation approach, when the true R is subcritical (R<1).** Results are based on simulations of outbreaks including a human-to-human transmission component (true R), and constant spillovers from a reservoir (α). The Bayesian sequential estimation method is applied to each of 50 simulated outbreak data. Iso-contours indicate the absolute discrepancy in R, estimated as the difference between the true R and the estimated median(R).

**
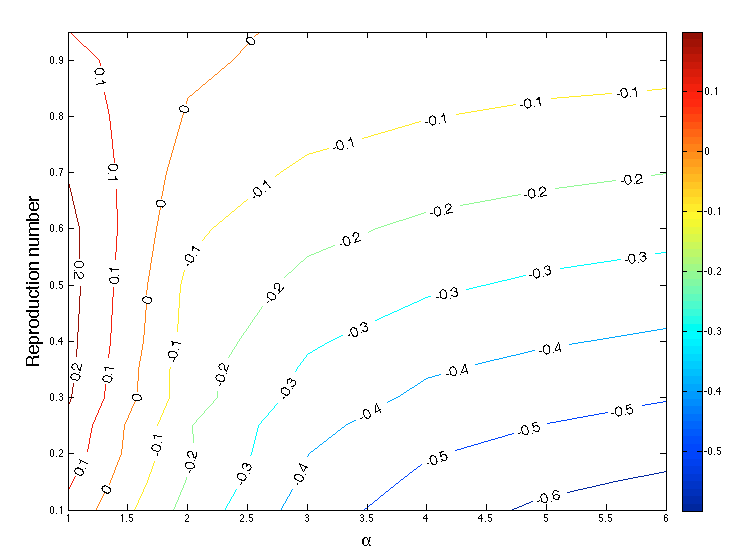
**

**Figure S4**: **R approximation based on the proportion of cases originating from human-to-human transmission.** Simulation studies indicate that the proportion of total cases arising from human-to-human transmissions is approximately equal to R, whenever R<1, and does not depend on the rate of spillover events α.

**
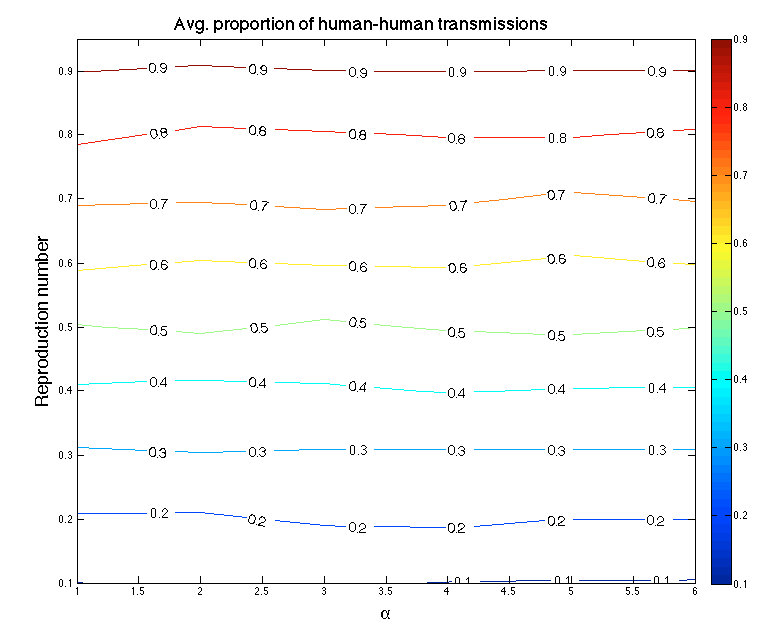
**

**Figure S5**. Predicted progression of cumulative laboratory-confirmed A/H7N9 cases in China (cases aggregated nationally), according to dates of symptoms onset in the absence of interventions (solid blue line). Dashed blue lines represent 95% confidence intervals. Predictions are based on an exponential model fit to the progression of reported cases from the end of February to April 6^th^, prior to live bird market closure, and using a negative binomial distribution to account for over-dispersion in case counts. Shown in red is the prediction of the model fit past April 6th. Black dots indicate the progression of reported A/H7N9 cases. Vertical dashed lines indicate the timing of the preemptive live bird market closures in Shanghai (April 6) and Zhejiang (April 15), respectively.

**
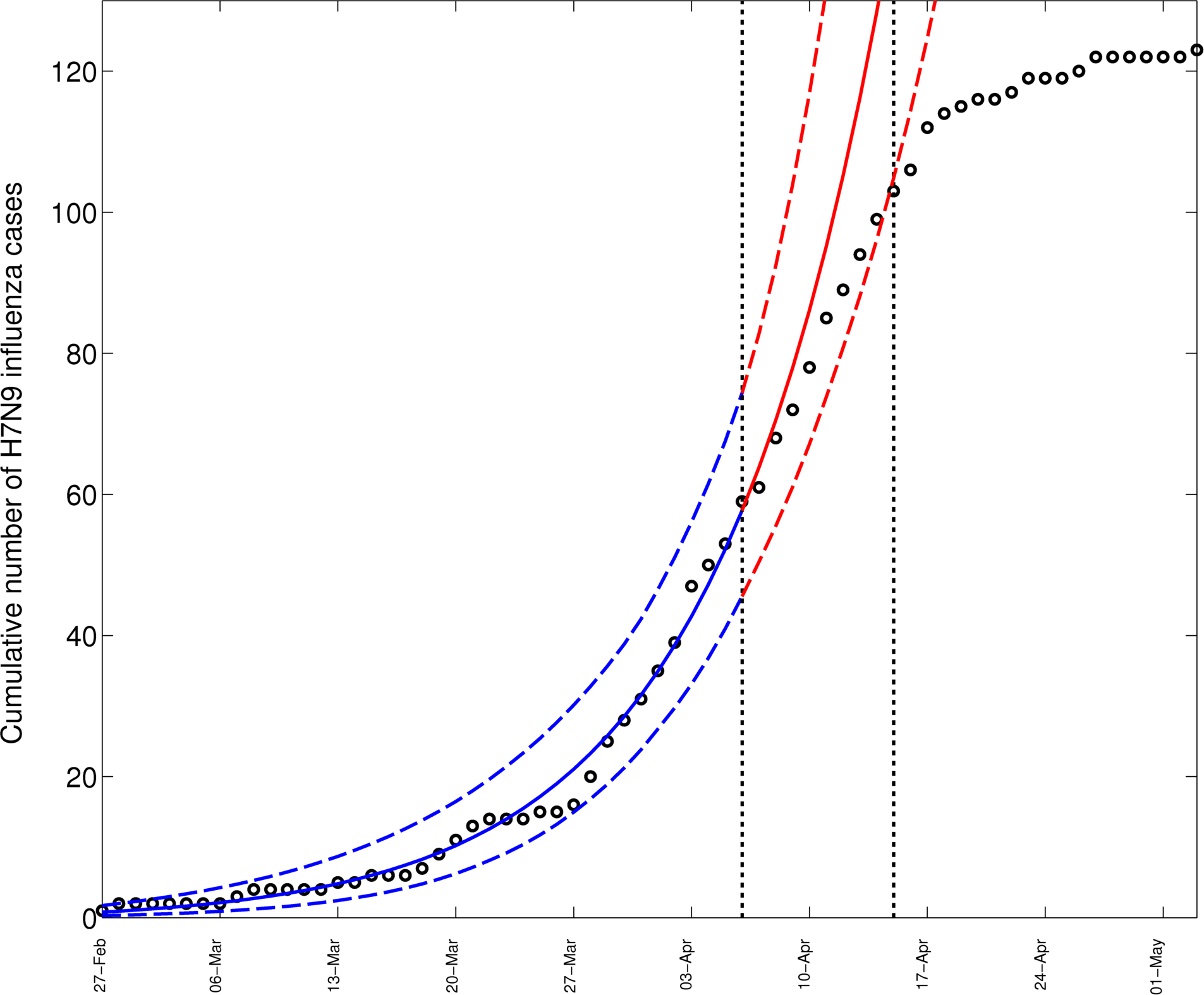
**

**Figure S6**. Slight changes in posterior distributions of R in Shanghai and Zheijiang when an extreme prior centered at R=1 is used.


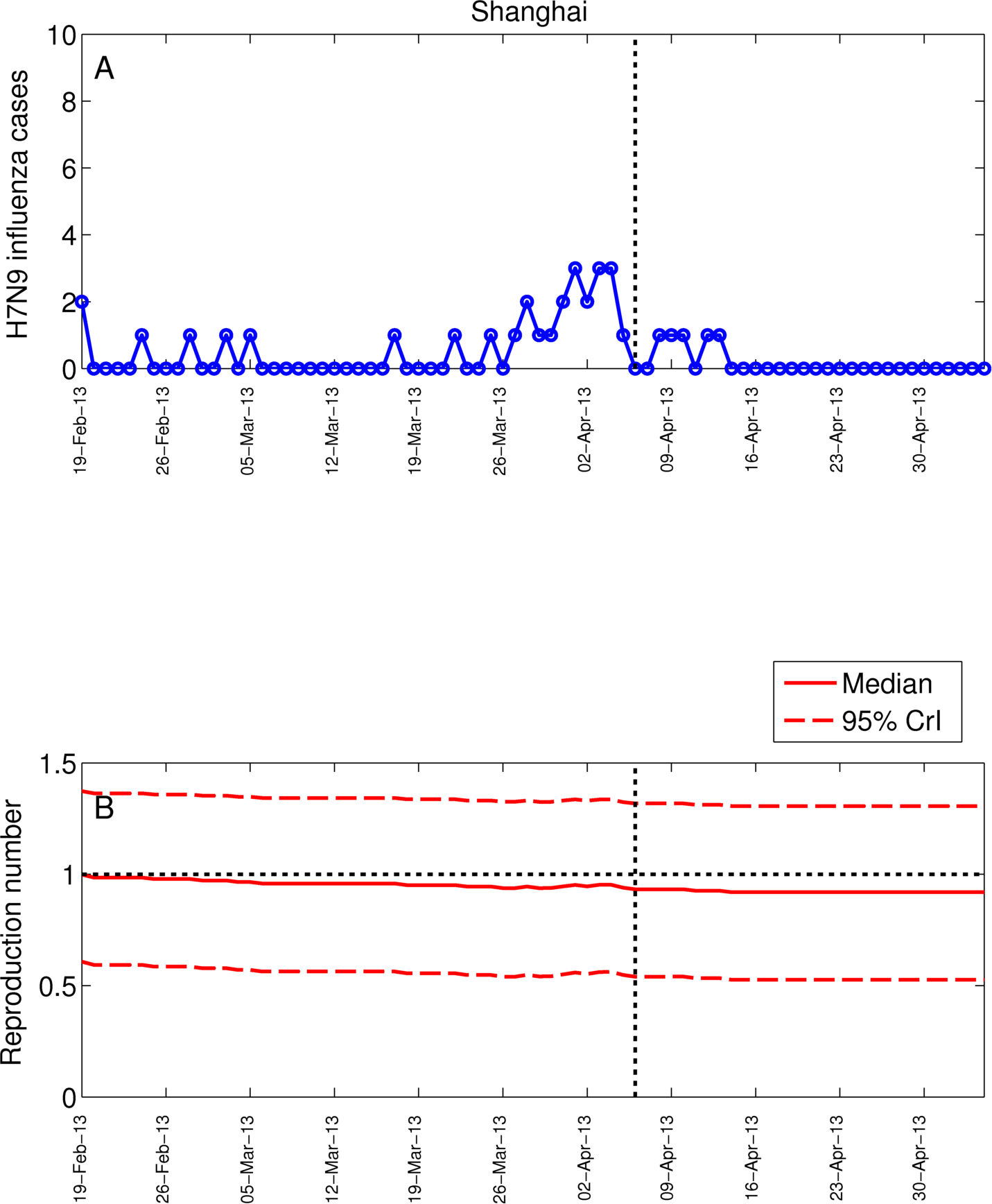


**
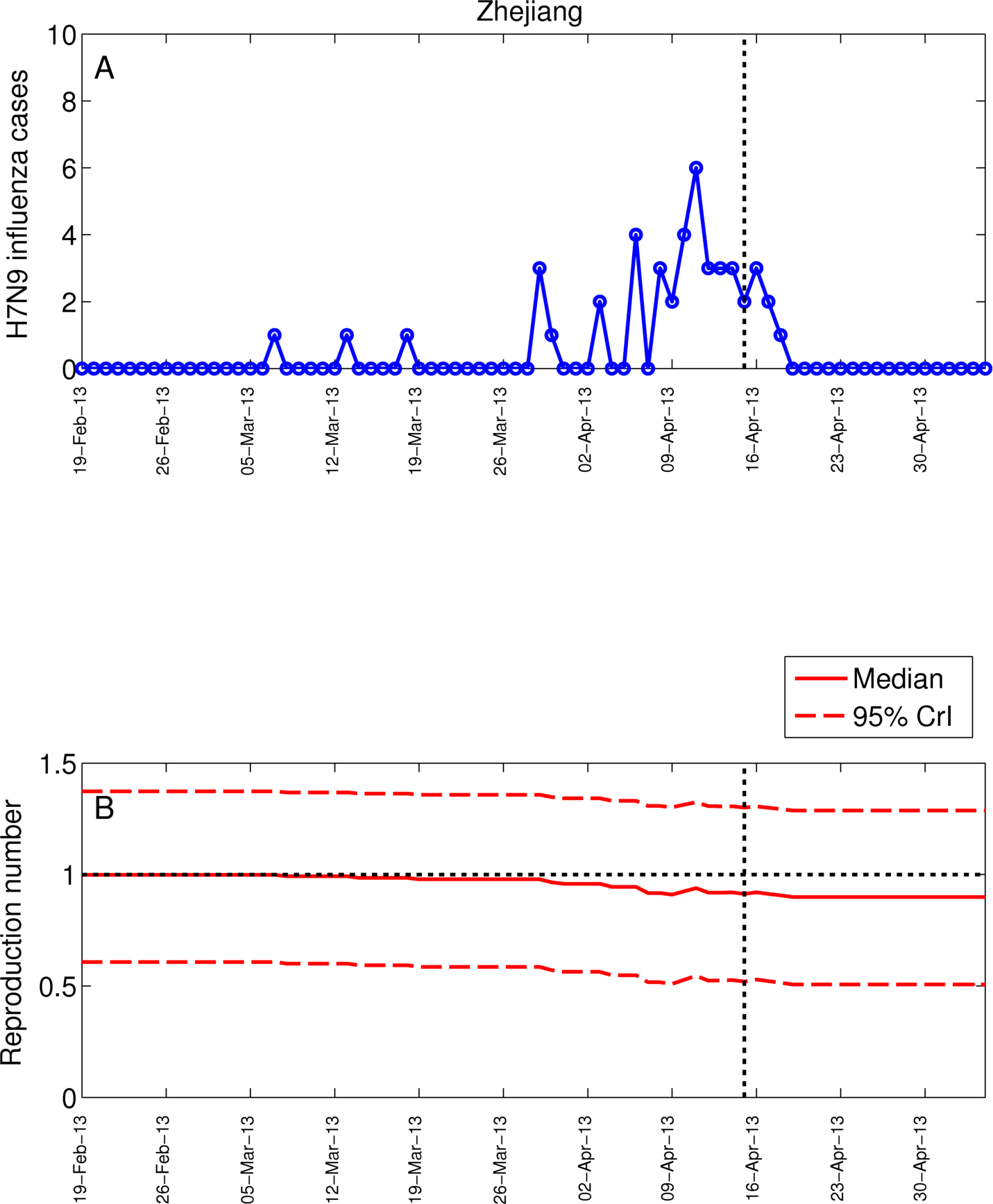
**

**Table S1**. Estimates and 95% credible intervals of the reproduction number for the A/H7N9 influenza outbreak in China based on the sequential Bayesian estimation SEIR method. In this sensitivity analysis, we assume a Negative Binomial distribution for the variance in daily incidence of A/H7N9 cases. Estimates are based on epidemiological data prior to the start of control interventions on April 6^th^, 2013.

| **Parameters** | **R estimate (95% CrI)** | |
| --- | --- | --- |
|  | **Zhejiang** | **Shanghai** |
| (k^-1^ = 3 days and ^-1^ = 3 days) | 0.15 (0.01, 0.48) | 0.16 (0.01,0.50) |
| (k^-1^ = 1.5 days and ^-1^ = 1.5 days) | 0.13 (0.005, 0.45) | 0.19 (0.01,0.52) |

**REFERENCES**
